# Protein Restriction in Metabolic Health: Lessons from Rodent Models

**DOI:** 10.3390/nu16020229

**Published:** 2024-01-10

**Authors:** Khuhee Na, Yoon Jung Park

**Affiliations:** 1 Department of Nutritional Science and Food Management, Ewha Womans University, Seoul 03760, Republic of Korea; 9942imkhyaa@naver.com; 2 Graduate Program in System Health Science and Engineering, Ewha Womans University, Seoul 03760, Republic of Korea

**Keywords:** macronutrients, protein restriction, FGF21, circadian rhythm, circadian clock

## Abstract

Consumption of protein-rich diets and supplements has been increasingly advocated by individuals seeking to optimize metabolic health and mitigate the effects of aging. Protein intake is postulated to support muscle mass retention and enhance longevity, underscoring its perceived benefits in age-related metabolic regulation. However, emerging evidence presents a paradox; while moderate protein consumption contributes to health maintenance, an excessive intake is associated with an elevated risk of chronic diseases, notably obesity and diabetes. Furthermore, recent studies suggest that reducing the ratio of protein intake to macronutrients improves metabolic parameters and extends lifespan. The aim of this study is to review the current evidence concerning the metabolic effects of protein-restricted diets and their potential mechanisms. Utilizing rodent models, investigations have revealed that protein-restricted diets exert a notable influence over food intake and energy consumption, ultimately leading to body weight loss, depending on the degree of dietary protein restriction. These phenotypic alterations are primarily mediated by the FGF21 signaling pathway, whose activation is likely regulated by ATF4 and the circadian clock. The evidence suggests that protein-restricted diets as an alternative approach to calorie-restricted regimes, particularly in overweight or obese adults. However, more research is needed to determine the optimal level of restriction, duration, and long-term effects of such interventions.

## 1. Introduction

Understanding an applicable and effective diet for improving chronic diseases in adults is a critical concern. Calorie restriction, defined as reducing food intake by 30% to 60% of ad libitum intake without causing malnutrition, is a commonly used dietary intervention due to its effects on weight loss and life extension [1]. However, adherence to calorie restriction is difficult to achieve in practice, particularly in middle-aged and older people [1]. There is a great deal of interest in developing a method that achieves similar results to calorie restriction without reducing food consumption [1]. An alternative approach could involve employing a strategy to modulate the macronutrient distribution in the diet. The ratio of macronutrients has a significant impact on lifespan [2,3]. The protein leverage hypothesis has recently highlighted the importance of protein ratios among macronutrients, suggesting that both absolute and relative amounts of protein preferentially influence our eating habits [4]. Hence, our focus is also on understanding the relationship between protein intake and human health.

Protein, a vital macronutrient, provides the essential amino acids required for the synthesis of proteins and other nitrogen components that are essential for the maintenance of the structural and functional systems of the human body [5]. Insufficient protein intake can lead to several health problems, including being underweight, preterm birth, growth retardation, and sarcopenia [6,7,8,9]. Adequate protein intake according to the life stage is therefore required due to its important role in the growth and maintenance of muscle mass and function [5,10]. Early-life protein intake is recognized as critical for growth and development [11,12,13]. Protein intake is particularly emphasized in elderly people due to its effects on the prevention and mitigation of sarcopenia, which is characterized by the progressive weakening of skeletal muscle [14,15]. Given its pivotal role, the use of protein and amino acid supplements, along with protein-rich meals, has become increasingly popular. In Korea, the current protein intake of adults exceeds the recommended levels [5]. High protein intakes within the energy ratio are known to induce greater satiety, reduce food consumption, and positively affect weight management due to their thermic effect [16,17]. However, these effects are mainly observed in short-term interventions, especially when combined with significant reductions in carbohydrate intake [18,19]. Conversely, studies suggest that excessive protein intake may increase the risk of chronic diseases, except in the elderly [20,21].

Several clinical and rodent studies have intriguingly demonstrated the beneficial effects of low-protein diets on metabolic health and longevity [2,22,23,24]. A study in which mice were given one of twenty-five different macronutrient diets ad libitum showed that the longest lifespan was associated with diets low in protein and high in carbohydrates [2]. Similarly, other research has shown that an ad libitum low-protein and high-carbohydrate diet provides similar benefits to calorie restriction in terms of levels of insulin, glucose, lipid, and HOMA, despite increased energy intake [22]. Yet, the mechanism remains unidentified, and the applicability of these findings to humans, based on current studies, remains uncertain. In this review, clinical data on protein intake will be distinguished by age group, and the results of rodent studies will be summarized and interpreted according to the level of protein restriction. The aim is to uncover whether dietary protein restriction would be metabolically beneficial using accumulated data.

## 2. The Effects of Protein Intake Rate on Adults and the Elderly

Recent epidemiologic and clinical evidence on the effects of various dietary protein levels in adults and the elderly are shown in Table 1. In adults, dietary protein has been shown to be effective for muscle health [25]. In a randomized controlled trial (RCT) study, a high-protein diet combined with exercise not only improved muscle power but also enhanced functional physical performance in middle-aged obese adults [25]. In the elderly, higher protein intake is required for their muscle health. Previous cross-sectional studies suggested that sarcopenia is associated with total protein intake [8,9]. Also, some RCT studies in elderly populations showed that overconsumption of protein (more than the RDA of 0.8 g/kg/day) or protein supplementation has beneficial effects in preventing sarcopenia and frailty [14,15]. The 1.5 g/kg/day protein intake group showed improvements in appendicular skeletal muscle mass (ASM) and gait speed compared to the lower-protein intake groups [14].

On the other hand, higher protein in the diet may be positively associated with several metabolic disorders [20,21,26]. A study using data from three prospective cohort studies showed that higher total protein intake was associated with an increased risk of type 2 diabetes [20]. In addition, a cross-sectional study of NAFLD patients found that those with a liver biopsy NAFLD activity score of 5–8 had a significantly higher daily protein intake than those with a NAFLD activity score of 0–4 [21]. High-protein diets have also been associated with increased mortality, particularly in adults aged 50–65 years who consume more than 20% of their total energy intake as protein [23]. Mortality from cancer and diabetes was four to five times higher in this group [23]. In middle age, dietary protein restriction may be effective in improving metabolic health, as suggested by some clinical data [23,24]. An interesting cross-sectional study of 6381 adults aged 50 years and older from The Third National Health and Nutrition Examination Survey, NHANES III reported that a low-protein diet is effective in middle age by reducing mortality from several causes [23]. In this study, participants were divided into a high-protein diet group (20% or more calories from protein), a moderate-protein diet group (10–19% of calories from protein), and a low-protein diet group (less than 10% of calories from protein) [23]. According to the predicted time till death graph, the low-protein diet had an effect in preventing all-cause and cancer mortality before the age of 66 years [23]. In another study of middle-aged overweight and mildly obese men, BMI, body weight, fat mass, and blood glucose intolerance were all decreased in the protein restriction group (7–9% protein diet for an average of 43 days) compared with the control diet group (~50% more energy from protein than the protein restriction group) [24]. Overall, the lower protein intake may reduce the risk of chronic diseases and extend lifespan in middle age. However, the protein ratio in the low-protein diets used in previous studies overlaps with the recommended protein intake rate of 7–20%. It is challenging to consider this a strict protein restriction. In addition, the exact mechanism behind these results remains unknown. Therefore, the mechanism needs to be elucidated by studies using strict protein-restricted dietary interventions. Most studies of low-protein diets in human models are cross-sectional, short-term, overfeeding diet studies, or studies using disease models [20,21,27,28,29,30,31,32].

## 3. Phenotypic Changes Resulting from Low Dietary Protein Intake Based on the Level of Restriction: Body Weight, Food Intake, and Energy Expenditure

**Figure 1:**
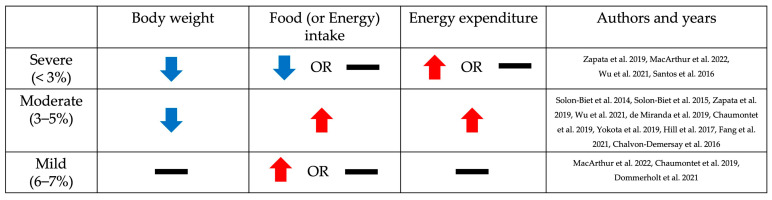
Changes in body weight, food (or energy) intake, and energy expenditure, depending on the level of dietary protein restriction in rodent studies. The trend of changes in body weight, food (or energy) intake, and energy expenditure according to the level of protein restriction compared to the control diet group is indicated by arrows and bars. Blue and red arrows indicate when the indicators decreased or increased in the protein-restricted group, respectively, and black bars indicate when there was no significant difference compared to the control diet group [2,22,27,28,29,30,31,32,34,35,36,37,38].

As shown in Figure 1, phenotypes such as body weight, food intake (or energy intake), and energy expenditure are affected by dietary protein restriction [27,28,29,30,31,32,33,34,35,36,37,38]. Body weight is decreased when fed severely and moderately protein-restricted diets (5% or less than 5% protein diets), while the group fed a mildly protein-restricted diet (6–7% protein diet) had no significant difference compared to the control diet group [27,28,29,30,31,32,33,34,35,36,37]. Food (or energy) intake is increased by moderately protein-restricted diets [2,22,29,32,35,38]. The severe protein restriction group had no significant difference in the amount of food (or energy) intake compared with the control protein diet group [27,28,29,30]. Interestingly, its level was decreased rather than increased in the severe protein restriction group compared with the control protein diet group [27,28,29]. On the other hand, in the mild protein restriction group, the food (or energy) intake was increased or had no significant difference compared with the control diet group [28,32,37]. Energy expenditure is also regulated by low-protein diets, especially 5% or less than 5% protein intake except 1% protein diets [22,27,29,32,35]. These data show that severe-to-moderate protein restriction induces weight loss. In particular, moderate protein restriction (3–5% protein diet) showed a weight loss effect despite an increase in food intake. Taken together, the mechanism of metabolic improvement may differ depending on the level of protein restriction.

### 3.1. Body Weight

Body weight was reduced in response to protein restriction (Table 2). Groups on severely and moderately protein-restricted diets (1–5% protein diet) had lower body weights compared to the control diet (12–20% protein diet) [27,28,29,30,31,32,33,34,35,36,37]. However, mild protein restriction (6–7% protein diet) did not result in any changes in body weight compared with the control diet (18% or 20% protein diet) [28,32,37].

### 3.2. Food (or Energy) Intake

In contrast to the decrease in body weight observed in the low-protein diet group, food or energy consumption tended to be increased to compensate for the dietary protein restriction (Table 2). According to a study in chronically ad libitum-fed mice, food intake was primarily controlled by the protein and carbohydrate content of the diet [2]. The dietary protein ratio was inversely correlated with the level of food intake [2]. It was consistent with results from most studies with moderate restriction (3–5%). The 5% protein diet group showed more food or energy intake than the control or high-protein diet group (14–60% protein diets) [2,22,27,29,35,38]. The studies employing low-protein diets of 3% or lower exhibited varying and inconclusive outcomes [32,33,34]. In an 8-week dietary intervention study using a female mouse model, a 3% low-protein diet resulted in increased energy intake [32]. However, in another study, male mice fed a 3% low-protein diet for 1 week showed no difference in food intake compared to the control group (20% protein diet) [33,34]. These different results may be due to sex and the duration of the dietary intervention. Moreover, severe protein restrictions such as a 1–2.5% protein diet did not significantly change the amount of food or energy intake and even resulted in less food or energy consumption compared to control diets (12%, 15%, 18%, and 20% protein diet, respectively) [27,28,29,30]. On the other hand, mice fed with a mildly protein-restricted diet (6–7% protein diet) did not show a significant change or increased food intake, compared to mice fed a control diet (18% or 20% protein diet) [28,32,37].

### 3.3. Energy Expenditure

Energy expenditure was increased in mice fed a low-protein diet, particularly in the moderate protein restriction group, not in the severe and mild protein restriction groups (Table 2). In moderate protein restriction (3–5%), energy expenditure was significantly higher on a low-protein diet compared to the control diet (15%, 20%, and 33% protein diet, respectively) [22,27,29,32,35]. On the other hand, there was no significant difference in the mild protein restriction compared to the control diet group (20% protein diet) [32]. One study that measured total energy expenditure by using the Weir equation showed that energy expenditure increased in the 3% protein restriction group, while there was no apparent change in the 6% protein diet group [32]. Severe protein restriction (<3%) shows inconsistent results [27,29]. In one study using an indirect comprehensive lab animal monitory system (CLAMS), energy expenditure initially increased in the 1% protein diet group compared with the 15% protein diet group but decreased significantly after 10 days of dietary intervention [27]. In another study using the Weir equation to measure energy expenditure, there were no significant differences in the 1% protein diet group compared to the control diet group (20% protein diet), but there was a significant increase in the 2.5% protein diet group [29].

## 4. Molecular Mechanisms Underlying Protein Restriction-Induced Metabolic Changes

### 4.1. Dietary Protein Restriction and FGF21

Fibroblast growth factor 21 (FGF21) is recognized as a major factor influencing the metabolic outcomes of dietary protein restriction [39]. It is a hormone that is mainly secreted by the liver [39]. In the absence of FGF21 activity, the effects of protein restriction described above did not occur [40,41]. In a study using an *Fgf21*-knockout mouse model, wild-type mice fed a low-protein diet for 2 weeks showed increased food intake and energy expenditure and decreased body weight compared to the control diet group [40]. However, the *Fgf21*-knockout mice did not show these phenotypic changes [40]. In another dietary intervention study using mice that were started on a chronic ad libitum low-protein diet at 3 months of age, they demonstrated that FGF21 was necessary for the effects of a low-protein diet, such as increased lifespan, reduced frailty, and altered metabolism [41]. Its mRNA expression increases in the liver when fed a protein-restricted diet, especially with a 5% or less low-protein diet (Table 3). In a study in which 25 diets with different ratios of protein, carbohydrate, and fat were randomly assigned to mice for 19 months, *Fgf21* mRNA expression in the liver and circulating plasma FGF21 levels were increased when protein intake was decreased [42]. Overall, FGF21 is required for the effects of dietary protein restriction.

### 4.2. A Key Mechanism Underlying FGF21-Mediated Metabolic Changes

**Figure 2:**
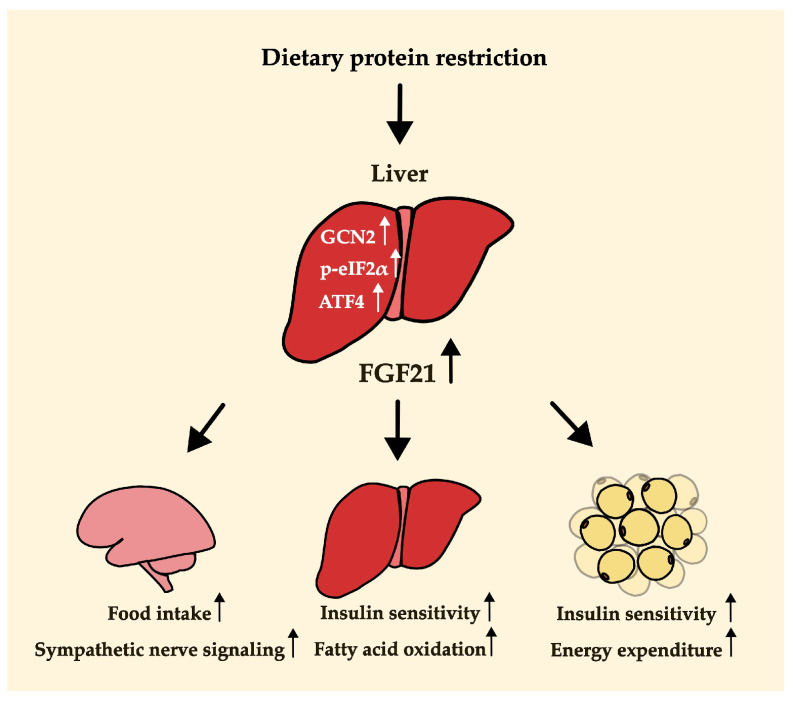
Potential mechanisms by which protein dilution alters body weight, food (or energy) intake, and energy expenditure. The arrow next to each word indicates an increase in activity.

It is known that an increase in the expression of *Fgf21* in a low-protein diet occurs via the GCN2-eIF2α pathway, which in turn increases its transcriptional factor, ATF4 [4,40,43]. Lack of dietary protein intake increases general control nonderepressible 2 (GCN2), which is involved in controlling amino acid metabolism by detecting nutrient deficiency in the liver and then inducing eukaryotic initiation factor 2 (eIF2α) phosphorylation [4,40,43]. The transcription factor, activating transcription factor 4 (ATF4), was increased by p-eIF2α, and it stimulates the expression of its target gene, *Fgf21* [43]. FGF21 produced in the liver is secreted by the liver and acts in the brain [44,45]. It binds to the complex of fibroblast growth factor receptor (FGFR) and beta-klotho in the brain, which is required for FGF21 to function in response to protein restriction [39,44]. The binding of FGF21 to this complex increases the expression level of corticotrophin-releasing factor (CRF) in the brain [39]. FGF21 may improve overall body metabolism by stimulating sympathetic nerve activity through increasing CRF [39,46]. A study using a rodent model found that the injection of CRF stimulated sympathetic flow to the brown adipose tissue (BAT) and thermogenesis [45]. Meanwhile, sympathetic nerve stimulation induced by FGF21 was completely blocked by the CRF receptor inhibitor [45]. CRF injection can also reduce body weight [47]. In addition, FGF21 upregulates the expression of neuropeptide Y (NPY), which is known to stimulate food intake in the hypothalamus [4]. Thus, the activity of FGF21 in the brain contributes to hyperphagia, body weight loss, and increased energy expenditure in a low-protein diet (Figure 2). FGF21 is also involved in the metabolism of peripheral tissues such as the liver and fat, as well as the central nervous system [39,45,48,49,50,51]. In the liver, increased FGF21 increases fatty acid oxidation and decreases lipogenesis and glucose production. It also increases energy expenditure and glucose uptake in the adipose tissue [39,45,48,49,50,51]. With the functions of FGF21 in the central nervous system and peripheral tissues, FGF21 may alleviate metabolic disorders such as obesity and diabetes [39,50,51,52].

### 4.3. A Potential New Mechanism Responsible for FGF21 Induction

**Figure 3:**
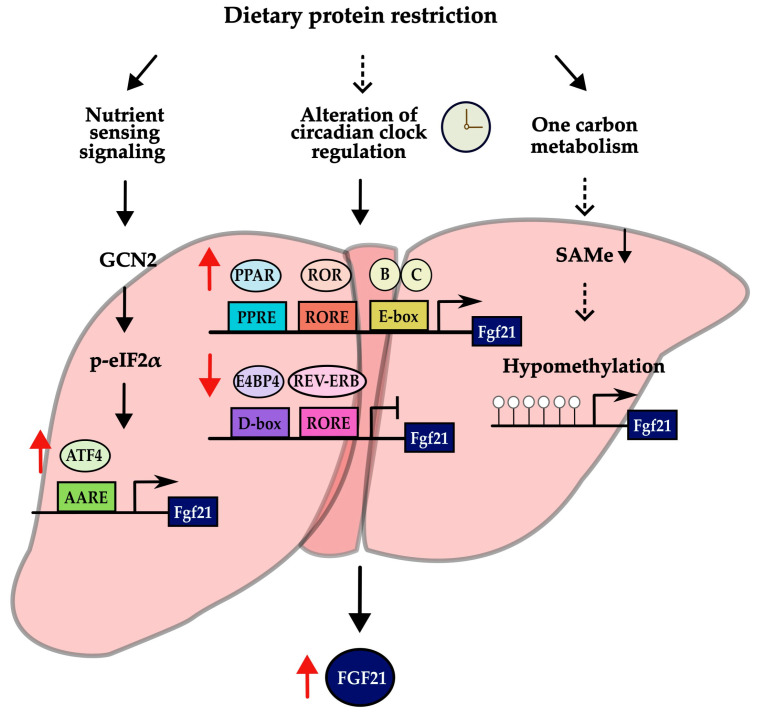
Possible mechanisms of inducing the expression of *Fgf21* in low-protein diet. Results with sufficient evidence are indicated by solid black arrows, while results with suggestive evidence are indicated by dashed black arrows. In addition, red arrows next to a transcriptional factor and FGF21 indicate an increase or decrease in its expression.

Regulation by the circadian clock may represent an alternative mechanism inducing the transcription of *Fgf21* in response to low protein intake, in addition to the GCN2–eIF2α–ATF4 pathway (Figure 3). The circadian rhythm has been actively studied in recent years because of its association with metabolic diseases. Studies have been conducted on the association with different dietary patterns [53,54,55,56,57,58]. According to a study on caloric restriction (CR), the circadian clock is responsible for regulating the rhythm of the ketogenic process induced by CR [58]. It was also found that circadian clock proteins are responsible for the transcriptional activity of *Fgf21*, which serves as a primary regulator of ketogenesis [58]. In addition, several lines of evidence have shown that the expression of circadian clock genes changes in response to the ratio of macronutrient intake in the diet [53,54,55,56,57]. These data support the potential involvement of the circadian clock in the upregulation of *Fgf21* expression during low protein intake.

The circadian rhythm is a biological process with a 24 h cycle [53]. This rhythm plays a critical role in glucose homeostasis, energy balance, the sleep–wake cycle, and hormone secretion [53]. It is controlled by clock genes in the body [59]. Clock genes can regulate many other genes through a transcription–translation feedback loop [60,61]. The most representative transcription factors of the core clock genes, CLOCK and BMAL1, form a heterodimer [60,61]. When this heterodimer rhythmically binds to an E-box element, it activates the transcription of clock-controlled genes (CCGs) encoding their own repressors, CRY (cryptochrome 1 and 2) and PER (period 1, 2 and 3) [60,61]. In a secondary loop, the REV-ERB (α and β) and ROR (α, β, and γ) proteins inhibit and activate *Bmal1* transcription, respectively [60,61]. REV-ERB represses *Bmal1* transcription by binding to RORE, whereas ROR activates its transcription [62]. REV-ERB is also stimulated by the heterodimer of CLOCK and BMAL1 that binds to the E-box [62].

The activity of the *Fgf21* promoter was dramatically increased by binding of the heterodimer of BMAL1 and CLOCK to the E-box on the promoter of *Fgf21* in the liver [58,63]. *Fgf21* transcription is also controlled by PPARα, REV-ERBα, RORα, and E4BP4 in the liver, which are the clock-controlled genes involved in the regulation of circadian rhythm [63,64,65,66,67]. *Fgf21* expression is repressed by E4BP4 and REV-ERBα and activated by PPARα and RORα [63,64,65,66,67]. Also, increased circulating FGF21 in a protein-restricted diet may depend on PPARα signaling [40]. *Pparα*-knockout mice demonstrated reduced levels of circulating FGF21 when subjected to a low-protein diet, in contrast to the wild-type mice fed with the same diet [40].

Taken together, these research data suggest that the circadian clock proteins may be involved in transcriptional regulation of *Fgf21* in the liver, which is a key factor for the effect of dietary protein restriction. Nevertheless, it remains elusive whether the hepatic circadian clock directly responds to dietary protein restriction. A noteworthy insight arises from a recent study that has unveiled a potential mechanism involving the hypomethylation of the *Fgf21* gene [68]. Methionine restriction led to the induction of *Fgf21*, as it resulted in the limitation of S-adenosylmethionine (SAMe), subsequently leading to DNA hypomethylation at the promoter region [68,69]. Nevertheless, there is currently a lack of knowledge regarding whether methylation levels decrease in response to a low-protein diet. Further research is essential to shed light on this important question.

## 5. Discussion

The existing evidence supporting the beneficial impact of protein-restricted diets on metabolism predominantly stems from studies conducted on young and middle-aged subjects, not on the elderly. To fully unlock the potential of these dietary strategies, it is crucial to gain a comprehensive understanding of the precise mechanisms governing the metabolic effects of protein restriction among macronutrients.

The primary question is which changes, such as protein amount, protein source, and amino acid composition, play a major role in the low-protein diet and cause such metabolic changes. According to papers, protein sources, including animal and plant proteins, influence metabolic health in middle-aged adults. According to a study of middle-aged Korean men, lower animal protein intake may be a beneficial factor in the management of metabolic syndrome [70]. In another study of Koreans over 40 years of age, animal protein intake was not significantly associated with dyslipidemia in men but was positively associated with the incidence of dyslipidemia in women according to several of its markers [71]. Increased consumption of red meat, an important source of essential amino acids among proteins, has been reported to increase the risk of chronic diseases, including cardiovascular disease (CVD), chronic kidney disease (CKD), and diabetes [72]. In addition, several studies showed that reducing specific amino acids, such as methionine or branched-chain amino acids (BCAAs), had beneficial effects on metabolic health [73,74,75,76]. Methionine restriction led to beneficial effects on metabolic health and longevity [73]. Research using high-fat diet-induced obesity has shown that methionine restriction ameliorates obesity and related metabolic problems [74]. Similarly, studies of BCAA restriction have shown metabolic improvements, including increased lifespan, reduced adiposity, weight loss, decreased fat mass, and improved glucose tolerance [75,76].

The results highlight that in adults, a population susceptible to the development of chronic disease, excess protein intake may increase the risk of chronic diseases and increase mortality from various causes, in contrast to the elderly (Table 1). Recent clinical studies suggest that a low-protein diet in middle age can potentially improve public health and extend life span [23,24]. However, the protein restrictions implemented in these studies overlap with the standard range of protein intake (7–20%). Therefore, it is premature to conclude that a low-protein diet inherently exerts a positive metabolic effect in humans, especially during middle age. Consequently, the critical step is to determine the optimal protein intake threshold that ameliorates chronic diseases and to devise a strategy applicable to humans using the accumulated rodent studies with a low-protein diet intervention.

Summarizing the phenotypic changes based on the extent of protein restriction, as shown in Figure 1, weight loss was observed in both severe and moderate protein restriction groups. However, food or energy intake and energy expenditure showed different patterns between severe and moderate protein restriction. This suggests the existence of different mechanisms in the metabolic improvement effects, including weight loss, depending on the level of protein restriction. One possibility, as illustrated in Figure 2 of this review, is that a low-protein diet induces an increase in FGF21, which stimulates nerves in the brain, thereby enhancing appetite and increasing energy expenditure in adipose tissue, ultimately leading to weight loss despite increased intake. The function of FGF21 in metabolic health in relation to protein intake levels was demonstrated by analyzing FGF21 polygenic scores for the risk of non-alcoholic fatty liver disease [77]. However, in the case of severe protein restriction, although the exact mechanism remains unclear, weight loss may have occurred due to reduced intake. Further research is needed to elucidate the differences in dietary intake based on the level of protein restriction and to understand how moderate protein restriction (3–5% protein diet) resulted in weight loss despite an increase in dietary intake.

Adjustment of the circadian clock is presented as one of the possible mechanisms (Figure 3). There is considerable evidence for an association between macronutrient intake and changes in the expression or rhythmicity of circadian clock genes [54,55,56,57] and an association between the intake pattern of macronutrients and susceptibility to chronic disease, depending on variants of circadian clock genes [78]. However, the specific effects of a low-protein diet on circadian clocks are not fully understood. Therefore, further research involving both animal and clinical interventions is needed to uncover clear mechanisms of the metabolic effects of a low-protein diet. For example, additional experiments are needed to confirm changes in the expression of circadian clock proteins or the recruitment of clock proteins to the promoter of *Fgf21* when protein is restricted.

In conclusion, a moderate low-protein diet, comprising 3–5% of total caloric intake, demonstrates the potential to enhance the transcriptional activity of *Fgf21*, leading to desirable anti-obesity outcomes, including significant weight loss. Thus, a low-protein diet may serve as a viable alternative to calorie-restricted regimens, particularly for individuals dealing with obesity or overweight conditions. Nonetheless, further research is imperative to fully elucidate the intricate mechanisms that underlie the metabolic enhancements and longevity-related effects associated with a low-protein diet.

## Figures and Tables

**Table 1:** Clinical studies demonstrating the effects of protein intake in adults and the elderly.

Life Stages	Initial Ages	Periods	Comparison Groups	Study Designs	Subject No.	Results	References
Adults (<65 years)	≥18 years	-	Patients with biopsy-proven NAFLD with an NAS of 5–8 vs. patients with an NAS of 0–4 ^1^	Cross- sectional	61	Disease activity and severity in patients with NAFLD was associated with a higher intake of dietary protein	[21]
		3 years	5% increase in intake of protein (14.3 ± 2.6% and 14.2 ± 1.9% energy intake from protein for women and men, respectively)	Prospective cohort	1254	Higher proportions of total protein in diet may be positively associated with higher increase in visceral adiposity index, especially among women	[26]
	≥24 years	-	Quintile of protein intake (14–22% energy of total protein)	Cross- sectional	205,802 ^2^	Greater intakes of total and animal protein were associated with a higher risk of type 2 diabetes	[20]
	50–65 years	-	LP diet (less than 10% of calories from proteins) vs. MP diet (10–19% of calories from proteins) vs. HP diet (20% or more of calories from proteins)	Cross- sectional	6381	LP diet had effect of preventing all-cause and cancer mortality before the age of 66 years old Cancer and diabetes mortality was four to five times higher in HP diet group aged 50–65	[23]
		12 weeks	Ex group ^3^ vs. Ex + HP group ^3^ vs. Control group ^3^	RCT	69	Exercise with a high-protein diet not only improved muscle power and exercise capacity but also enhanced functional physical performance in middle-aged obese adults	[25]
	52–53 years	43 days (mean)	LP diet (7–9% of protein diet) vs. MP diet (~50% more protein diet)	RCT	38	BMI, body weight, fat mass, and blood glucose intolerance were all decreased in overweight and mildly obese ^4^ males fed LP diet	[24]
Elderly (≥65 years)	≥65 years	-	LP diet (less than 10% of calories from proteins) vs. MP diet (10–19% of calories from proteins) vs. HP diet (20% or more of calories from proteins)	Cross- sectional	6381	MP and HP diet had effect of preventing all-cause and cancer mortality in those over 66 years old	[23]
	70–85 years	12 weeks	0.8, 1.2, or 1.5 g/kg/day group	RCT	120	In 1.5 g/kg/d group, ASM and gait speed (m/s) were more improved than other groups	[14]
	74.8 ± 5.9 years	-	Sarcopenia (70.2 ± 20.2 g protein/day) vs. non-sarcopenia (85 ± 28.3 g protein/day)	Cross- sectional	331	Sarcopenia was associated with total protein intakes	[9]
	87 years (mean)	-	Robust (78 g protein/day) vs. Probable sarcopenia (72 g protein/day) vs. Sarcopenia (66 g protein/day)	Cross- sectional	126	Sarcopenia was associated with total protein intakes	[8]

NAFLD: non-alcoholic fatty liver disease; LP: low protein; MP: moderate protein; HP: high protein; RCT: randomized controlled trial; BMI: body mass index; ASM: appendicular skeletal muscle mass. ^1^ The NAFLD activity score (NAS) is defined as the unweighted sum of the scores for steatosis (0–3), lobular inflammation (0–3), and ballooning (0–2), thus ranging from 0 to 8. ^2^ NHS (The Nurses’ Health Study) 72,992 + NHS II (Nurses’ Health Study II) 92,088 + HPFS (Health Professionals Follow-up Study) 40,722. ^3^ Ex group (12 weeks of exercise training) vs. Ex + HP group (12 weeks of exercise training + high-protein diet intervention (1.6 g protein/kg/day) vs. Control group (Maintain their lifestyle for 12 weeks). ^4^ Overweight and mild obesity: baseline BMI ~30 kg/m^2^.

**Table 2:** Rodent studies demonstrating changes in body weight, food (or energy) intake, and energy expenditure with dietary protein restriction.

Level of Protein Restriction	Low- Protein Diets	Control Diets	Rodent Models (Strain, Sex, Initial Age)	Periods	Body Weight	Food Intake (FI) or Energy Intake (EI)	Indirect Energy Expenditure	References
Severe (<3%)	1%	15%	Sprague Dawley rats Male 7 weeks old	3 weeks	Decreased	EI: No significant difference initially and decreased from day 8 of dietary intervention	Initially increased, but its level significantly decreased from day 10 of dietary intervention	[27]
		20%	C57BL/6N mice Male 12 weeks old	12 weeks	Decreased	EI: Decreased	No significant difference	[29]
	2%	12%	Swiss Webster mice Male 2–3 months old	5 weeks	Decreased	FI: No significant difference	-	[30]
		18%	B6D2F1 mice Male 16 weeks old	1 week	Decreased	FI: Decreased	-	[28]
	2.5%	20%	C57BL/6N mice Male 12 weeks old	12 weeks	Decreased	EI: No significant difference	Increased	[29]
Moderate (3–5%)	3%	12%	Swiss mice Female 6 weeks old	5–6 weeks	Decreased	-	-	[31]
		20%	ICR mice Male 8 weeks old	1 week	Decreased	FI: No significant difference	-	[34]
			BALB/c mice Female 9 weeks old	8 weeks	Decreased	EI: Increased	Increased	[32]
	5%	14–60%	C57BL/6 mice Male and female 3 weeks old	24 weeks	-	FI: Increased	-	[2]
		14.5%	C57BL/6 mice Male 8 weeks old	3 weeks	-	FI: Increased	-	[38]
		15%	Sprague Dawley rats Male 6 weeks old	3 weeks	Decreased	EI: Increased	Increased	[27]
		20%	C57BL/6J mice Male ~3 months old	6 weeks	Decreased	FI: Increased	Increased	[35]
			C57BL/6N mice Male 12 weeks old	12 weeks	Decreased	EI: Increased	Increased	[29]
			C57BL/6J mice Male ~3 months old	76 weeks	Decreased	-	-	[36]
		33%	C57BL/6J mice Male 8 weeks old	8 weeks	-	EI and FI: Increased	Increased	[22]
Mild (6–7%)	6%	18%	B6D2F1 mice Male 16 weeks old	1 week	No significant difference	FI: No significant difference	-	[28]
		20%	BALB/c mice Female 9 weeks old	8 weeks	No significant difference	EI: No significant difference	No significant difference	[32]
	7%	20%	C57BL6/J mice Male 18 months old, 3 months old	12 weeks	No significant difference	FI: Increased	-	[37]

**Table 3:** Rodent studies demonstrating the alteration of the hepatic *Fgf21* expression by dietary protein restriction.

Level of Protein Restriction	Low Protein Diets	Control Diets	Rodent Models (Strain, Sex, Initial Age)	Periods	*Fgf21*	References
Severe (<3%)	1%	20%	C57BL/6N mice Male 12 weeks old	12 weeks	Increased	[29]
	2%	18%	B6D2F1 mice Male 16 weeks old	1 week	Increased	[28]
	2.5%	20%	C57BL/6N mice Male 12 weeks old	12 weeks	Increased	[29]
Moderate (3–5%)	3%	20%	BALB/c mice Female 9 weeks old	8 weeks	Increased	[32]
	5%	14.5%	C57BL/6 mice Male 8 weeks old	3 weeks	Increased	[38]
	5%	20%	C57BL/6J mice Male ~3 months old	6 weeks	Increased	[35]
	5%	20%	C57BL/6N mice Male 12 weeks old	12 weeks	Increased	[29]
	6%	18%	B6D2F1 mice Male 16 weeks old	1 week	Increased	[28]
Mild (6–7%)	6%	20%	BALB/c mice Female 9 weeks old	8 weeks	No significant difference	[32]
	7%	20%	C57BL6/J mice Male 18 months old (old), 3 months old (young)	12 weeks	Old: increased Young: No significant difference	[37]
